# Green Infrastructure Design Influences Communities of Urban Soil Bacteria

**DOI:** 10.3389/fmicb.2019.00982

**Published:** 2019-05-16

**Authors:** Jessica Lee Joyner, Jordan Kerwin, Maha Deeb, George Lozefski, Bharath Prithiviraj, Anna Paltseva, John McLaughlin, Peter Groffman, Zhongqi Cheng, Theodore R. Muth

**Affiliations:** ^1^Department of Biological Sciences, Brooklyn College of The City University of New York, Brooklyn, NY, United States; ^2^Department of Biology, Georgia State University, Atlanta, Georgia; ^3^Department of Earth and Environmental Sciences, Brooklyn College of The City University of New York, Brooklyn, NY, United States; ^4^Advanced Science Research Center at the Graduate Center of the City University of New York, New York, NY, United States; ^5^Graduate Center of The City University of New York (CUNY), New York, NY, United States; ^6^New York City Department of Environmental Protection, Flushing, NY, United States

**Keywords:** soil, green infrastructure (GI), bacteria, urban ecosystem, microbial ecology

## Abstract

The importance of natural ecosystem processes is often overlooked in urban areas. Green Infrastructure (GI) features have been constructed in urban areas as elements to capture and treat excess urban runoff while providing a range of ancillary benefits, e.g., ecosystem processes mediated by microorganisms that improve air and water quality, in addition to the associations with plant and tree rhizospheres. The objective of this study was to characterize the bacterial community and diversity in engineered soils (Technosols) of five types of GI in New York City; vegetated swales, right of way bioswales (ROWB; including street-side infiltration systems and enhanced tree pits), and an urban forest. The design of ROWB GI features directly connects with the road to manage street runoff, which can increase the Technosol saturation and exposure to urban contaminants washed from the street and carried into the GI feature. This GI design specifically accommodates dramatic pulses of water that influence the bacterial community composition and diversity through the selective pressure of contaminants or by disturbance. The ROWB had the highest biodiversity, but no significant correlation with levels of soil organic matter and microbially-mediated biogeochemical functions. Another important biogeochemical parameter for soil bacterial communities is pH, which influenced the bacterial community composition, consistent with studies in non-urban soils. Bacterial community composition in GI features showed signs of anthropogenic disturbance, including exposure to animal feces and chemical contaminants, such as petroleum products. Results suggest the overall design and management of GI features with a channeled connection with street runoff, such as ROWB, have a comprehensive effect on soil parameters (particularly organic matter) and the bacterial community. One key consideration for future assessments of GI microbial community would be to determine the source of organic matter and elucidate the relationship between vegetation, Technosol, and bacteria in the designed GI features.

## Introduction

Urban centers are rapidly growing at the sacrifice of natural or green spaces. Approximately 82% of the population in North America resides in an urban area with 400–1000 people per square kilometer (McGranahan et al., 2005). This increase in population density contributes to greater generation of pollutants and waste, which amplifies the potential for elevated exposure to pollutants for residents. Globally, population estimates project that approximately 68% of the world’s population will reside in urban areas by 2050 (UNDESA, 2017, 2018). This growing threat to environmental resources and public health is a driving force for communities to adopt sustainable practices that improve and maintain urban environmental health. A solution has been to redesign the urban landscape with the objective of multifaceted benefits for the urban center, such as green infrastructure (GI). A strong motivation specific for green infrastructure is to mitigate disruptions in the local watershed hydrology caused by impervious surfaces and reduced natural vegetation (Shuster et al., 2005; Nowak and Greenfield, 2012; Capps et al., 2016). GI features are designed using a combination of natural and constructed materials to capture urban runoff and facilitate other functions provided by natural systems (Tzoulas et al., 2007; Morel et al., 2014). Cities around the world have begun to improve existing infrastructure using GI to manage storm water runoff, enhance air quality, and provide additional social and economic benefits (Benedict and McMahon, 2006; Flynn and Traver, 2013; Rakhshandehroo et al., 2015; Kumar and Hundal, 2016; Connop et al., 2016; Gill et al., 2017). In New York City (NYC, United States), a detailed GI plan includes a range of designs (Table 1) to achieve ambitious goals for urban runoff and storm water capture while providing benefits related to pollutant removal, aesthetic enhancement, climate modification and other ecosystem services (Pires, 2004; NYC Environmental Protection, 2012).

**Table 1:** Summary of GI designs present in the 22 sites sampled in New York City, NY.

GI design		Definition (Green infrastructure annual report 2012)
ROWB		Right of Way Bioswale: ROWB are constructed within sidewalks and are adjacent to the road– they are designed to catch street and sidewalk runoff by being positioned upstream of existing sewer catch basins; runoff is then directed to the vegetation in the system (NYC Environmental Protection, 2012)
	•*ETP*	Enhanced Tree Pit: Contains a top layer of Technosol along with either glass, gravel as belowground storage for excess water (NYC Environmental Protection, 2010)
	•*SSIS*	Street-side Infiltration Swale: Larger surface area than an ETP; contains one layer of top Technosol, and lacks belowground water storage. (NYC Environmental Protection, 2010)
VS		Vegetated Swale: Areas of vegetation that are located in expansive areas such as parking lots etc. that vary in size and shape
UF		Urban Forest: Dense collection of trees in an urban area. ∼ our site was at Alley Pond Park established in 1935 and is an irregular shaped area of land with dense canopy

*All GI were constructed with Technosol from the same source with a high sand content (∼70–85%).*

A range of ecosystem services are provided by GI features, though optimizing these services relies on the selection of locally-appropriate and resilient plant cover as well as the use of engineered soil (Technosols; Deeb et al., 2016) to balance water infiltration and nutrient cycling processes for specific urban landscapes (Lehman, 2006; Macías and Camps Arbestain, 2010; NYC Environmental Protection, 2012; Gkorezis et al., 2016; Takaijudin et al., 2016; Li et al., 2016). Environmental and structural characteristics of GI features, particularly soil texture, greatly influence local microclimatic conditions by regulating the availability of surface water films for soil microbes and water retention for plants and meso – macrofauna (Huang et al., 2002; Cosandey et al., 2003; Jia and Conrad, 2009; Ball, 2013). Additionally, variations in organic matter, pH, and salts, can influence soil microbial community composition, and function (Cosandey et al., 2003; Ng et al., 2016; Wang et al., 2018). Soil microbial communities maintain essential roles that are responsible for nutrient cycling processes which support plant growth and other ecosystem services of GI features (Wall, 2004; Young and Crawford, 2004; Hostetler et al., 2011). Furthermore, biodiversity, particularly within the soil microbiome, has been found to be important for maintaining overall ecosystem function and resilience (Allison and Martiny, 2008; Reese et al., 2015; Gkorezis et al., 2016; Neilson et al., 2017). With a vast majority of Earth’s biological diversity contained within belowground soil ecosystems, it is important to understand the anthropogenic impacts on soil through land use development (Roesch et al., 2007; Delmont et al., 2011; Wang et al., 2018).

Analysis of the microbial communities in GI features is fundamental to understanding the role of the microbial community and as a guide for future GI designs. The microbial community of Technosols can be an indicator of the nature and extent of anthropogenic impact on GI features, including pollutants in urban runoff. Studies across a range of locations and soil types show that the Phyla Acidobacteria, Actinobacteria, and Proteobacteria regularly dominating the soil microbiome, including in urban soils (Fierer and Jackson, 2006; McGuire et al., 2013; Huot et al., 2017). These Phyla provide a baseline for evaluating the composition of bacterial communities in Technosols. In GI features, anthropogenic impacts such as exposure to petroleum products, heavy metals, salt, animal feces, pesticides, fertilizers, and garbage in urban runoff can shift abundances of bacteria within the Technosol microbiome (Sauvé et al., 1997; Marcin et al., 2013; Delgado-Balbuena et al., 2016; Adeniji et al., 2017). These changes can be attributed to introductions of atypical soil microbiota as well as enrichment of specific, indigenous bacteria. For example, gram-negative bacteria typically increase in soils contaminated with petroleum (MacNaughton et al., 1999); specifically, the genus *Pseudomonas* contributes to this increase because species within this genus are able to metabolize petroleum products (Barathi and Vasudevan, 2001; Rahman et al., 2002; Nikhil et al., 2013). Some gram-positive bacteria, i.e., *Micrococcus*, have also been observed to increase in the presence of petroleum contamination (Nikhil et al., 2013). Biological contamination through animal feces can introduce *Clostridium* and *Ruminococccus* species and, more broadly, the Phyla Firmicutes, Bacteroidetes, and Actinobacteria into the soil (Handl et al., 2011). Knowing whether these, and other bacteria, are found in GI features is important because it can help to guide urban planning for the purposes of improving urban biodiversity or bioremediation, such as influencing the location, design, construction of GI features. Previous studies have characterized correlations between anthropogenic impacts on soil microbial communities, however, limited research has explored the dynamics influencing the Technosol microbial communities in GI features (Mukherjee et al., 2017).

The objective of this study was to describe the bacterial community of Technosols in GI features, which differ in their design for managing urban runoff infiltration and retention in NYC. Focal bacterial groups were selected because of their essential role in nutrient cycling and the degradation of pollutants. Relationships between bacterial communities, composition and diversity, with soil physical properties and biogeochemical parameters were evaluated by comparing results with data from a companion study conducted on the same sites (Deeb et al., 2018).

## Materials and Methods

### GI Site Description

**FIGURE 1:**
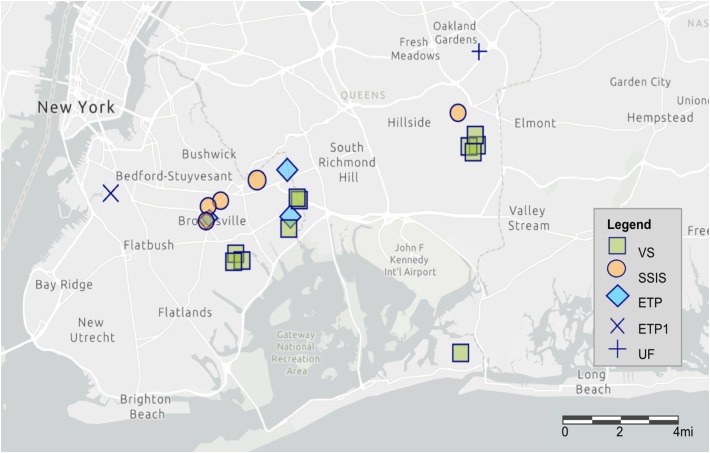
Map of the 22 sampled GI sites through Brooklyn and Queens boroughs of New York City, NY. The map was created using ArcGIS^®^software by Esri with the light gray canvas basemap.

Samples were collected from Technosols of GI features in two New York City Boroughs within the Jamaica Bay, NY watershed. Sites (*N* = 22) were selected to represent the variety of NYC GI designs for right-of-way bioswales (ROWB); enhanced tree pits (ETP; *N* = 5) and street-side infiltration swales (SSIS; *N* = 5), as well as vegetated swales (VS; *N* = 11) and an urban forest (UF; *N* = 1) (Figure 1 and Table 1). All GI features were constructed with Technosol from the same source with a high sand content (70–85%). Modifications to the road drainage system (i.e., curb cuts, inlet modifications, and catch basin modifications) directly channel runoff to ROWB (NYC Environmental Protection, 2010, 2012). Both ETP and SSIS are prototypes of bioswales and were constructed within sidewalks adjacent to streets, directly connected to street drainage. The ETP design is a smaller surface area than the SSIS and has an integrated runoff detention chamber. The VS are vegetated areas of variable size and shape, constructed in open areas (e.g., parking lots, parks) to provide surface, soil, and gravel storage for infiltration of urban runoff. The area of ETP are typically 9.3 m^2^ (6.1 m by 1.5 m), with a 0.6 m engineered soil layer over 0.6 m of gravel, recycled glass, or storage chambers. The surface area of SSIS are typically 18.6 m^2^ (12.2 m by 1.5 m) and do not contain a storage layer with gravel, recycled glass, or storage chambers. One ETP site (GI.0.14, ETP1) had a 0.3 m wide gravel strip along the curb instead of soil and a larger surface area (∼19 m^2^). The VS sample sites had an average surface area of 269.8 m^2^ and were most commonly located at large street intersections and parking lots. One VS site served as an urban reference site (GI.11.25). The UF site was sampled as a reference of a non-Technosol urban soil and was an irregularly shaped forest stand of native hardwood trees (oak and hickory) established in 1935.

The area of impervious urban surface creating runoff water to be managed by a GI feature differed across the GI sites in this study. ETP sites had 156–411 m^2^ and SSIS sites had 207–1847 m^2^ of impervious area contributing potential urban runoff. Since VS sites had unstructured design criteria including the size of the GI feature, the area of impervious surfaces each VS site served varied from 73 to 7606 m^2^.

The initial plant cover for all designs was a combination of herbaceous perennials, grasses, and trees. The herbaceous plant cover at all sites included American boneset (*Eupatorium perfoliatium*), New England aster (*Aster novae angiliae* syn. *Symphyotrichum novae-angliae*), and oxeye sunflower (*Heliospsis helianthoides*). The grasses at all sites were switch grass (*Panicum virgatum*) and Virginia wild rye (*Elymus virginicus*). Trees planted varied by site and were selected from the following resilient species – black gum (*Nyssa sylvatica*), sweet gum (*Liquidambar styraciflua*), shadblow (*Amelanchier canadensis*), and swamp white oak (*Quercus bicolor*). At the time of sampling, vegetation cover was generally present and ROWB sites were consistent with the planned selection of species (Supplementary Figure 1).

### Sample Collection

GI sites were sampled over a period of 48 h in July, 2016. No rain was recorded while sampling or for 48 h prior and the July air ranged from 22 to 30°C. Multiple surface samples were collected at each GI site from random locations within the site (distributed throughout the center, periphery, etc.) at a depth of 0–10 cm. All surface samples from a single GI site were pooled into a larger collection barrel and mixed thoroughly as a collective representation of that site. Final samples were then collected as a subset of the larger composite collection barrel representing each site and stored at 5°C until DNA extraction (within 24 h of collection). Bacterial DNA was extracted from soil (0.25 g) using the MoBio PowerSoil DNA Isolation kit (Qiagen MoBio, Carlsbad, CA, United States). The DNA was stored at -20°C until it was shipped overnight for sequencing. Isolated DNA was amplified for the 16S rRNA gene, V1-V3 region (for details see Supplementary Description) then sequenced using Illumina MiSeq (Molecular Research Lab, Shallowater, TX, United States).

### Biogeochemical Parameters

In a companion study (Deeb et al., 2018), GI site samples were analyzed for a series of soil chemical variables; organic carbon (C-org), total nitrogen (N-total), pH, salts, total petroleum hydrocarbons (TPH), Pb, Zn as well as moisture content (MC) and texture. A suite of additional measurements for biogeochemical parameters (microbial biomass carbon and nitrogen content, potential net nitrogen mineralization and nitrification, microbial respiration, and denitrification potential) were also completed (Supplementary Table 1).

### Microbiome Sequence Analysis

Sequence data were received as paired forward and reverse reads with adapters removed. Subsequent processing was accomplished using the Quantitative Insights Into Microbial Ecology analysis tool (QIIME v1.9.1; Caporaso et al., 2010). Sample specific barcodes and poor quality sections of sequences were trimmed away and remaining sequences were filtered to remove overall poor-quality reads: minimum length of 200 basepairs, average quality score of >25, no more than 6 homopolymers or ambiguous bases. The resulting sequences were then clustered, using the Usearch algorithm (Edgar, 2010), into operational taxonomic units (OTUs, minimum cluster size of 4 for filtering) and were aligned with FastTree then assigned a taxonomic identification at 97% threshold for PyNAST alignment using the Greengenes v13.8 database. Non-bacterial (e.g., mitochondria, chloroplasts, and Archaea) sequences are common in 16S amplicon libraries; therefore, they were identified and filtered from the GI sample sequences. Throughout processing the sequence data, the sequence average for each sample remained about 50,000 sequences (Supplementary Table 2). Final bacterial communities were normalized by rarefying to the lowest number of the sample sequence counts, 44,702 sequences (rarefaction curves, Supplementary Figure 2) Sequence counts of replicates were averaged to better represent the bacterial community of each GI design (R Core Team analysis package Phyloseq; McMurdie and Holmes, 2012).

Several biodiversity metrics were calculated using the normalized sequence data. The top 20 Orders were calculated and represented along with the combined local contribution to beta diversity (LCBD, using default Hellinger dissimilarity distance) using the MicrobiomeSeq R package (Legendre and De Cáceres, 2013; Ssekagiri et al., 2018). The LCBD was qualitatively used to describe the bacteria community profiles, with scale of LCBD indicating a sample’s similarity (small) to other samples or difference (larger). Alpha diversity represents bacterial diversity within each sample and GI design; richness of unique organisms is presented as the observed OTUs and estimated richness of OTUs (Chao1), while the Shannon diversity index accounts for unique OTU richness and abundance. Beta diversity represents the bacterial diversity between GI samples, calculated with a Bray-Curtis and Unifrac (weighted and unweighted) distance matrix of the bacterial community similarities represented by a principal coordinate analysis, PCoA (Phyloseq). A PERMANOVA was completed to test the significance of cluster of samples, specifically by GI design.

The biogeochemical parameters measured (Deeb et al., 2018) were compared to the bacterial community to investigate relationships between community composition and function (Ssekagiri et al., 2018). Predominant bacterial Orders related to biogeochemical parameters were determined with linear correlations; significance determined with *p*-values of less than 0.05 adjusted for multiple comparisons by false discovery rate (fdr; Williams et al., 2014). Additionally, the most abundant bacteria at the Order level (Orders with greater than a total of 50 sequences for all samples) were evaluated for associations with biogeochemical factors common to pollutants using ANOVAs followed by Tukey HSD tests.

Results from this study were compared with a previous sampling of natural (not-engineered) urban surface soils from non-GI features in NYC (Huot et al., 2017). The sequence data from these urban surface soil samples also targeted the bacterial community using the V1–V3 16SrRNA region and were sequenced using Illumnia MiSeq by the same laboratory (Molecular Research Lab, Shallowater, TX, United States). The primary GI sequence data (22 sequenced samples averaged by location, *N* = 17) and the urban surface soil sequence data (28 sequenced soils averaged by location, *N* = 9) were re-analyzed as a single batch. The analysis methods were the same as described above except that new OTU clusters were identified using the Uclust algorithm to reduce computational demand (Edgar, 2010). Sequences for all samples were normalized to control for sequencing variation between samples as well as machine runs for the different data sets. Differential abundances for bacterial Orders were evaluated by averaging the number of sequences for the Orders at each site then comparing the proportion of the urban surface soil bacterial community to the proportion within the Technosol bacterial community. Significant differences were determined by completing a Welch’s *t*-test for unbalanced samples on the average sequence abundance of each site for each Order between urban surface soil and Technosols.

## Results

### Biogeochemical Parameters

Microbial biomass C and N and metabolic functioning (potential net N mineralization and nitrification, microbial respiration, denitrification potential) were all positively correlated with C-org, N-total, pH, MC, TPH, and watershed contributing area (Deeb et al., 2018) (Table 2). Denitrification potential and microbial biomass C and N were higher in ETP followed by SSIS then VS (Table 3). The pH was markedly lower in the UF (4.1) than in the VS, ETP and SSIS (6.7, 6.4, 6.39, respectively). All Technosols had similar soil texture with more than 70% sand (Table 2). Levels of heavy metals (Pb, Zn, Ni) and TPH at GI sites did not exceed regulatory or published levels for polluted soils (Deeb et al., 2018). However, ETP had significantly (ANOVA, *p* < 0.05) greater levels of TPH, Pb, and Zn than SSIS and VS, which did not differ significantly. ETP sites had higher levels of organic carbon that likely drove higher levels of microbial biomass and activity in these sites compared to the SSIS and VS sites. For most biogeochemical parameters evaluated, SSIS had higher levels of activity than VS sites, but these differences were not always statistically significant.

**Table 2:** Green infrastructure physical and chemical characteristics.

Type	OM ^∗∗∗^ (mg kg^−1^)	pH ns	salts ns (mg kg^−1^)	TPH ^∗∗∗^ (mg kg^−1^)	Sand % ^∗∗∗^	Clay % ^∗∗^	N_Total_ % ^∗∗∗^	SOC % ^∗∗∗^	Pb ^∗∗∗^ (mg kg^−1^)	Zn ^∗∗∗^ (mg kg^−1^)	Ni ns (mg kg^−1^)	MC % ^∗∗∗^
ETP	12.03 ± 1.08^a^	6.45 ± 0.06	151.65 ± 14.72	990 ± 156.69^a^	71.52 ± 1.74^a^	12.48 ± 1.77^a^	0.33 ± 0.03^a^	6.51 ± 0.53^a^	115.50 ± 17.68^a^	352.37 ± 47.26^a^	71.20 ± 3.05	0.31 ± 0.04^a^
SSIS	8.57 ± 0.91^a^	6.39 ± 0.07	115.86 ± 11.54	588 ± 76.37^b^	71.80 ± 2.13^a^	18.57 ± 1.26^b^	0.35 ± 0.08^a^	4.17 ± 0.42^b^	67.88 ± 13.72^b^	202.36 ± 17.26^b^	68.97 ± 2.79	0.19 ± 0.02^b^
UF	10.27 ± 0.87^a^	4.09 ± 0.01	188.00 ± 9.29	190 ± 00.01^c^	71.67 ± 5.60^a^	14.09 ± 3.57^a^	0.60 ± 0.07^b^	6.19 ± 0.77^a^	146.00 ± 00.01^a^	113.00 ± 00.01^a^	64.00 ± 0.01	0.23 ± 0.01^c^
VS	4.94 ± 0.86^b^	6.64 ± 1.16	119.29 ± 20.76	420.90 ± 73.27^b^	82.73 ± 14.40^b^	12.27 ± 2.14^a^	0.15 ± 0.03^c^	2.95 ± 0.51^c^	57.64 ± 10.03^b^	188.30 ± 32.78^b^	73.42 ± 12.78	0.12 ± 0.02^d^

*Values are mean ± SE (n = 15 except for VS n = 33 and UF n = 3). Significant code: ^∗∗∗^P < 0.0001; ^∗∗^P < 0.001; ^∗^P < 0.05; ns = non-significant. Significant differences (P < 0.05) are indicated by different letters (Deeb et al., 2018).*

**Table 3:** Green infrastructure microbial function characteristics.

Type	Microbial biomass C^∗∗∗^ (μg C g^−1^_dry soil_)	Microbial biomass N^∗∗∗^ (μg N g^−1^_dry soil_)	Respiration^∗^ (μg C g^−1^_dry soil_ day^−1^)	Mineralization^∗∗∗^ (μg N g^−1^_dry soil_ day^−1^)	Nitrification^∗∗∗^ (μg N_g_^−1^_dry soil_ day^−1^)	DEA^∗∗∗^ (μg N_g_^−1^_dry soil_ day^−1^)
ETP	923.82 ± 141.89^a^	77.79 ± 6.25^a^	25.92 ± 5.18^a^	1.28 ± 0.50^a^	0.68 ± 0.21^a^	2.97 ± 0.66^a^
SSIS	633.70 ± 128.96^b^	53.13 ± 6.34^b^	30.96 ± 8.79^a^	0.67 ± 0.22^ab^	0.76 ± 0.20^a^	3.01 ± 0.72^a^
UF	521.93 ± 00.01^c^	101.22 ± 3.87^a^	30.50 ± 0.01^a^	0.58 ± 0.01^b^	0.56 ± 0.05^b^	0.93 ± 0.05^b^
VS	375.13 ± 65.30^c^	35.17 ± 6.12^c^	13.82 ± 2.41^b^	0.15 ± 0.03^c^	0.22 ± 0.04^c^	0.56 ± 0.10^c^

*Values are mean ± SE (n = 15 except for VS n = 33 and UF n = 3). Significant code: ^∗∗∗^P < 0.0001; ^∗∗^P < 0.001; ^∗^P < 0.05; ns non-significant. Significant differences (P < 0.05) are indicated by different letters (Deeb et al., 2018).*

### Microbiome Profile

**FIGURE 2:**
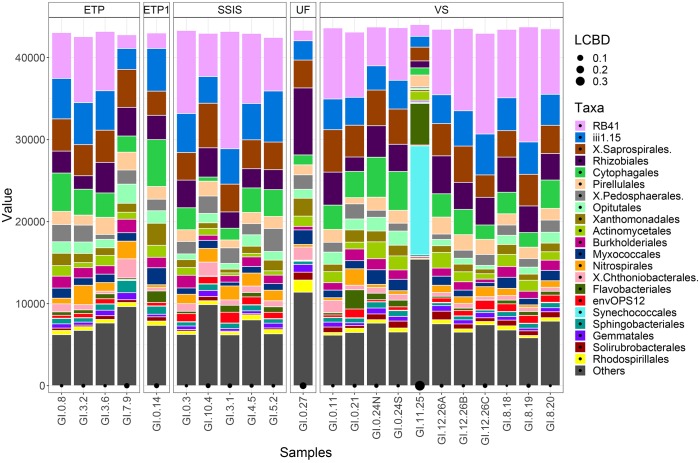
Bacterial communities were normalized to the lowest sequence count of the samples (44,702). The taxa sequence abundances are grouped by the different GI designs: Enhanced Tree Pit (ETP), Modified Enhanced Tree Pit (GI.0.14; ETP1), Street-Side Infiltration System (SSIS), Vegetated Street (VS), and Urban Forest (GI.0.27; UF). Taxa of the bacteria communities are represented by the top 20 Orders with the remaining Orders clustered into the Other category. Local contribution to beta diversity (LCBD) indicates the uniqueness of a community with the black circles along the x-axis, size corresponds to the scaled difference.

Technosol bacterial communities varied with GI design, most notably with communities in VS sites distinct from those in the ETP and SSIS. Specific community composition of bacterial Orders was only significantly unique for the UF site as well as for the VS urban reference site, GI.11.25 (Figure 2); the UF site was more similar to the VS than to other GI designs.

**FIGURE 3:**
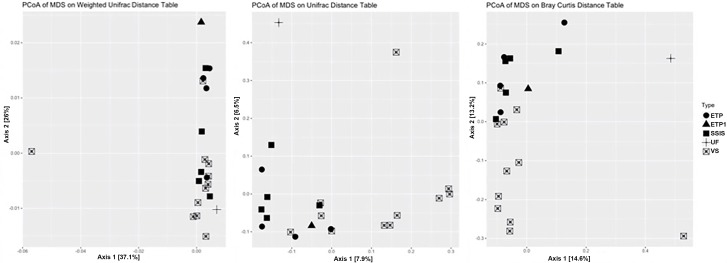
The beta diversity comparison of bacterial communities. PCoA analysis plots for Weighted Unifrac (left, incorporates phylogenetic relatedness and abundance within communities), Unweighted Unifrac (center; incorporates phylogenetic relatedness within communities), and Bray-Curtis (right) distance matrices.

The GI bacterial communities all had levels of diversity in a range that is similar to the diversity associated with non-urban soils (Fierer and Jackson, 2006; McGuire et al., 2013; Ramirez et al., 2014; Delgado-Baquerizo et al., 2018), and phylogenetically similar organisms were present in all the GI sites. Once normalized for sequencing depth (44,702 sequences for each sample), and removing outliers (ETP1, UF, and Urban Reference-GI.11.25), VS had significantly greater observed and estimated (Chao1) richness than SSIS (Table 4, Tukey HSD, *p* = 0.009). The SSIS had an average of 8722 OTUs and a Shannon diversity index of 7.6792 H′, the ETP had an average of 9143 OTUs and 7.8133 H′, and VS had an average of 9688 OTUs and 7.9383 H′. The UF had 7528 OTUs and 7.5868 H′ (Table 4 and Supplementary Figure 3). The bacterial Orders observed in all Technosol samples, from greatest to least, were RB41 and iii1-15 (both Phylum Acidobacteria), Saprospirales, Rhizobiales, Cytophagales, Pirellulales, Pedosphaerales, Opitutales, Xanthomonadales, and Actinomycetales (Figure 2). On average there were fewer Pedosphaerales (ANOVA, *p* = 0.015) and more Actinomycetales (ANOVA, *p* = 0.007) in VS than in the other designs. No other abundant nor prevalent bacterial Order significantly varied in abundance between GI designs. GI designs had unique bacterial community beta diversity when considering both the phylogenetic relatedness of bacteria present as well as the abundance of each taxon. The variation across the samples is best explained by the Weighted Unifrac but the clustering observed is not significant (PERMANOVA, *p* = 0.105), while the Unifrac and Bray Curtis distance matrices had significant clustering by GI design (PERMANOVA, *p* = 0.001 and *p* = 0.007, respectively, Figure 3). While GI designs had similar communities, within the VS GI design, the urban reference site bacterial community was unlike the other VS sites in the PCoA analysis. One key difference was the high relative abundance of Synechoccales, which was rare in the other VS sites.

**Table 4:** Alpha biodiversity calculated for samples normalized to the lowest sequenced sample data (44,702 sequences) and for then averaged for GI Design with 95% confidence intervals.

	GI site				GI design		
Sample	Observed OTUs	Predicted OTUs (Chao1)	Shannon Index	Type	Observed OTUs	Predicted OTUs (Chao1)	Shannon Index (H′)
GI.0.27	7528	9736	7.5868	UF	–	–	–

GI.3.2	8801	12762	7.6468	ETP	9143 ± 754.4	12852 ± 1084.58	7.8133 ± 0.2185
GI.3.6	9697	13615	7.9664				
GI.7.9	8699	11978	7.7683				
GI.0.8	9374	13051	7.8715				

GI.0.14	8800	11473	7.9340	ETP1	–	–	–

GI.3.1	7828	19289	7.3368	SSIS	8722 ± 643.7	13776 ± 3877.13	7.6792 ± 0.2530
GI.4.5	8802	11727	7.7623				
GI.5.2	8934	13123	7.6685				
GI.10.4	8873	12255	7.8661				
GI.0.3	9171	12484	7.7623				

GI.8.18	10111	14512	8.0070	VS *(excluding GI.11.25)*	9688 ± 322.6	13412 ± 628.16	7.9383 ± 0.1162
GI.8.19	9434	13838	7.6567				
GI.8.20	10260	14411	8.0256				
GI.11.25	5102	9127	5.1780				
GI.12.26A	10089	14040	8.1209				
GI.12.26B	9563	13095	7.9644				
GI.12.26C	9042	12825	7.7023				
GI.0.11	9550	12120	7.9287				
GI.0.21	8967	12017	7.8150				
GI.0.24N	9880	13628	8.0736				
GI.0.24S	9988	13633	8.0890				

There were significant trends between specific biogeochemical parameters and the top 10 Orders of bacteria in the GI sites (Supplementary Figure 4), however, none of the biogeochemical parameters were consistently correlated across bacterial Orders or GI design. Chromatales and A21b were among the 10 abundant Orders across all GI designs. Chromatales had significant positive correlation (*p* < 0.05) with the MC of ETP and SSIS but not VS, while A21b also significantly correlated (*p* < 0.05) positively with MC, but only for VS. OM content was high across all GI sites, but only significant (*p* < 0.05) twice – positively with Anaerolineales in VS and negatively with Neisseriales in ETP.

**FIGURE 4:**
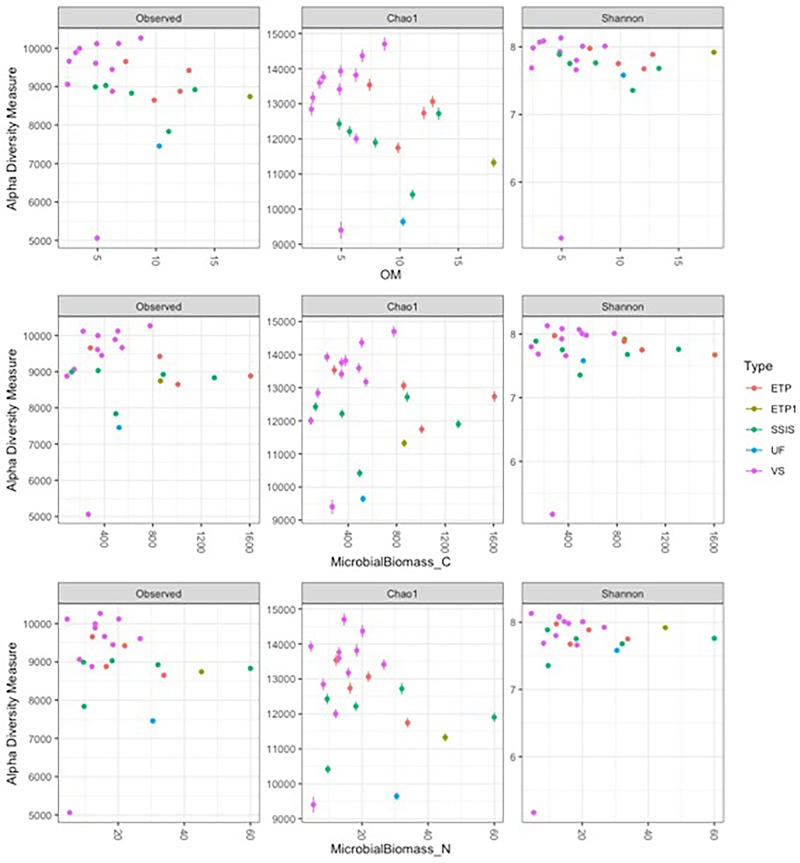
Alpha diversity metrics of the rarefied sample dataset with key biogeochemical parameters. ETP1 and UF are not replicated so there is no regression model but they are included for comparison. Observed is the number of unique OTUs within samples, Chao is the estimated richness of OTUs in a sample, Shannon are diversity indices that balance the richness (Observed unique OTUs) with the abundance of each OTU. Within the GI sites, OM is the organic matter measured and Microbial Biomass C and N are the C and N within the living portion of the soil sampled.

The biogeochemical parameters did not significantly influence diversity patterns in the communities, only the GI design had a significant influence on community diversity (ANOVA for GI design interaction with biogeochemical parameter, *p* < 0.05 for GI design alone). As OM increased there was slight trend for microbial diversity to increase in VS and decrease in both SSIS and ETP (Figure 4). Similar to the trend with general OM, with greater levels of microbial biomass C and N, VS sites had greater diversity in contrast to the decrease in diversity for ETP and SSIS (Figure 4). As the pH shifted toward neutral or basic, SSIS increased in diversity and VS decreased (Supplementary Figure 5). The salt concentration within the Technosols had a wide range and higher concentrations correlated with a decrease in abundance and an overall decrease in diversity for VS and SSIS designs, while ETP design increased in diversity (Supplementary Figure 5).

**FIGURE 5:**
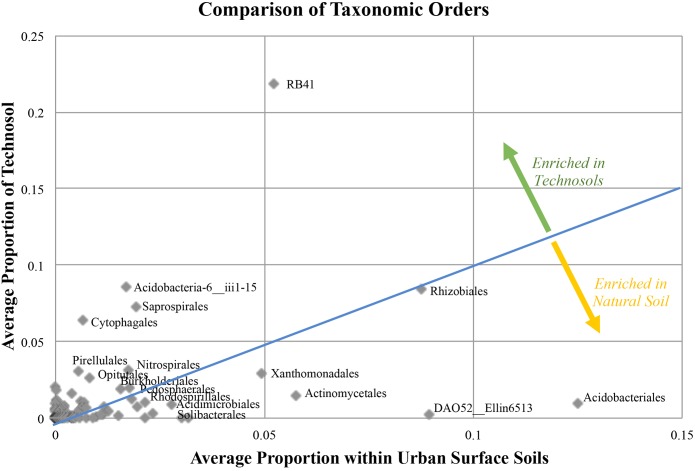
A summary of how the bacterial community differs in urban soils. Natural (*N* = 9) urban surface soil still has anthropogenic influence, but is not purposefully manufactured like Technosols in GI (*N* = 17). Equal proportion or representation in the soils is represented by the x = y line, values represented above this line indicate the Order was more prevalent in Technosols and values below the line represent Orders more prevalent in urban natural soils.

When Technosols were compared with natural urban surface soils, some differences in community composition were apparent after normalizing for sequence variation (3,567 sequences for each sample). Of the 163 bacterial Orders present in both groups of samples (Figure 5); 56 Orders had a greater abundance in Technosols than in the urban surface soils. Most notable in both sets of samples was the Class Chloracidobacteria, Order RB41 (Welch’s *t*-test, *p* = 0.1415). The Order Acidobacteriales had a slightly greater abundance in the urban surface soils than in the Technosols (Welch’s *t*-test, *p* = 0.0548). Technosols and urban surface soils had differences in the most abundant (top 10%) Orders in the bacterial community. The two groups of samples only shared 6 of 25 Orders while few abundant Orders were significantly different (Table 5). Actinomycetales was in the top 10% of both Technosols and urban surface soils and were, similarly, abundant (Welch’s *t*-test, *p* = 0.1693). Rhizobiales were equally represented and just under 10% of the average bacterial community for both urban surface soils and Technosols. Bacillales were included in the top 10% of Technosols and not in the urban surface soils, but the difference was not statistically significant. For Technosols, Pirellulales, and Synechococcales were in the top 10% and in significantly greater abundance than in the urban surface soils. (Welch’s *t*-test = 0.0278 and 0.0255, respectively). While the less described bacteria DA052_Ellin6513 and ABS-6_NA were significantly more abundant in urban surface soils than Technosols.

**Table 5:** A compiled list of bacterial Orders comprising the top 10% average abundance within Technosols (^†^, *N* = 17, with sub-samples of sites averaged) and natural urban soils (^§^, *N* = 9 with surface horizons averaged by site).

Taxonomy: Class__Order	Technosol (Ave sequences)	Natural (Ave sequences)	*p*-value
[Chloracidobacteria]__RB41	531.12^†^	124.03^§^	0.1415
Acidobacteria-6__iii1-15	205.45^†^	44.55	0.7304
Alphaproteobacteria__Rhizobiales	170.04^†^	202.79^§^	0.3819
[Saprospirae]__[Saprospirales]	167.24^†^	44.84	0.0510
Cytophagia__Cytophagales	141.64^†^	18.72	0.1425
Nitrospira__Nitrospirales	81.84^†^	45.85^§^	0.6822
Planctomycetia__Pirellulales	69.78^†^	15.33	0.0278^∗^
Opitutae__Opitutales	60.30^†^	20.60	0.1671
Synechococcophycideae__Synechococcales	59.40^†^	0.00	0.0255^∗^
Gammaproteobacteria__Xanthomonadales	59.08^†^	124.89^§^	0.3085
[Pedosphaerae]__[Pedosphaerales]	47.04^†^	48.51^§^	0.1423
Betaproteobacteria__Burkholderiales	45.28^†^	38.71	0.2018
Anaerolineae__envOPS12	44.80^†^	0.17	0.1782
Bacilli__Bacillales	42.25^†^	10.67	0.4173
Bacteroidia__Bacteroidales	33.80^†^	1.00	0.2886
Actinobacteria__Actinomycetales	32.67^†^	137.80^§^	0.1693
Deltaproteobacteria__Myxococcales	25.69	45.71^§^	0.0061^∗^
[Spartobacteria]__[Chthoniobacterales]	24.09	57.90^§^	0.8958
Alphaproteobacteria__Rhodospirillales	18.65	66.19^§^	0.1832
Thermoleophilia__Solirubrobacterales	13.59	47.24^§^	0.2772
Acidobacteriia__Acidobacteriales	13.01	280.49^§^	0.1739
Acidimicrobiia__Acidimicrobiales	9.26	68.04^§^	0.0547
Solibacteres__Solibacterales	0.46	76.32^§^	0.1483
DA052__Ellin6513	0.41	198.46^§^	0.0201^∗^
ABS-6__NA	0.00	78.70^§^	0.0087^∗^

*Significant difference determined by a Welch t-test with p < 0.05 (^∗^).*

Soil bacteria are known to contribute to bioremediation of environmental pollutants, therefore identifying bacteria in GI can help to evaluate the bioremediation potential of GI designs (Gadd, 2007; Glick, 2010; Ojuederie and Babalola, 2017; Santos Dos and Maranho, 2018). Bioremediation of TPH has been associated with a few key Orders: Pseudomonadales, Actinomycetales, Flavobacteriales, Bacillales, and Clostridiales (Paul et al., 2005; Jiao et al., 2016). Of these Orders, only Actinomycetales varied with GI design; there was significantly lower abundance in SSIS than in VS (Tukey’s HSD, *p* = 0.007) and no difference with ETP sites. These patterns are consistent with levels of TPH, which were significantly higher in ETP sites than in SSIS and VS sites. The levels of TPH were low enough to not present a human health risk; furthermore, at this low level there was limited influence on the bacterial community with only Actinomycetales correlated with TPH levels. Additionally, GI features have evidence of enteric bacteria contamination, which is likely from the introduction of animal feces. The Orders Bacillales and Clostridiales are often considered enteric bacteria and were common in GI sites. Other enteric bacteria are within the Phyla Firmicutes, Bacteroides, and Actinobacteria. These Phyla, including the identified lower taxa, were present in GI sites with only minor shifts in abundance across GI designs. Actinomycetales (within the Phylum of Actinobacteria) were less abundant in SSIS and VS (significant only for VS, Tukey’s HSD, *p* = 0.02). Results for animal introduction of bacteria are also consistent with lower levels of microbial biomass in SSIS and VS than in ETP features.

## Discussion

Design of the GI features influenced the bacterial community composition within the soil, with the VS having distinct bacterial communities compared to the ETP and SSIS features. The ETP and SSIS are more excessively engineered features, with direct connections to urban runoff from streets. While VS are constructed with variable size, shape, and integration with urban runoff management. Bacterial communities are also known to differ with varied pH (Fierer, 2017; Gill et al., 2017), however, in this study pH did not vary enough between the GI designs to identify a significant effect on bacterial diversity nor composition. Core similarities between the bacterial communities of ETP and SSIS were likely influenced by the consistent texture (high sand content) of the Technosols, area, and increased connectivity.

The differences in bacterial community composition, with the VS features having the most distinct communities, raise questions about relationships between microbial community structure and function. ETP sites had higher levels of organic carbon that drove higher levels of microbial biomass and metabolic functioning in these sites compared to the SSIS and VS sites. For most biogeochemical variables, SSIS had higher levels than VS sites, but these differences were not always statistically significant. Interestingly, organic matter, microbial biomass, and soil moisture, which should be strong drivers of community composition, do not solely explain differences between the ETP, SSIS, and VS features. There is great uncertainty about urban ecosystem functioning due to multiple unknowns (i.e., local urban development, vehicle or pedestrian traffic, or vegetation survival, etc.) associated with construction and design of GIs (Kaye et al., 2006). Still, these and other GI features have been shown to support high levels of nitrogen cycling activities important to water quality (denitrification) and plant production (nitrogen fixation) (Pickett et al., 2008; Pataki et al., 2011; Morse et al., 2017).

Technosols had relatively high levels of OM compared to non-urban soils (Vasenev and Kuzyakov, 2018); thus, in this study, microbes were not likely competing for carbon resources. Consequently, higher OM did not lead to higher diversity in the ETP, although it did lead to higher microbial biomass (Deeb et al., 2018). This suggests that influences on bacterial diversity may differ from the influence on overall microbial biomass. For VS, there was a positive relationship between microbial biomass C and N content and diversity; yet, there was a negative relationship for ETP and no relationship for SSIS. These results suggest a key difference between VS and ROWB is in the designed connectivity to urban runoff. This connectivity combined with size of the GI design, may be strong influences on microbial community structure and function.

The relationships between OM, bacterial community composition and biogeochemical function are particularly interesting for bioremediation potential of GI features. GI sites with relatively high OM also had higher TPH concentrations: TPH was the highest at ETP followed by SSIS and VS, likely associated with the sorption of TPH on OM (Deeb et al., 2018). ETP had the greatest variation and amount of TPH, with the trend of sites with higher levels of TPH had a decline in bacterial richness. Increases in the Phyla Xanthamonadales, Rhizobiales, and Rhodobacterials are associated with the degradation of TPH contaminants such as polycyclic aromatic hydrocarbons (PAH) (Delgado-Balbuena et al., 2016). In our study, Xanthamonadales and Rhizobiales were in the top 10% of both Technosol and natural urban surface soils, however, there was no significant difference in abundance between GI designs. The consistent prevalence of both Xanthamonadales and Rhizobiales may be due to their strong association with surface vegetation, which can also correlate with OM. At all GI sites there was surface vegetation. However, inconsistent survival of tree species and herbaceous plants make further research necessary draw to conclusions about the relationship between vegetation type and amount, OM levels, microbial community and the dynamics of organic contaminants such as TPH.

Similar to previous descriptions of the urban soil microbiome, Acidobacteria, Actinobacteria, and Proteobacteria are among the most abundant bacterial Phyla of Technosols (Yan et al., 2016; Supplementary Figure 6). The bacterial communities of Technosols revealed similar diversity to previously studied natural soils. This is likely to be important for their ability to sustain ecosystem functions and is encouraging given that Technosols are not entirely naturally derived. The Orders Bacillales and Clostridiales are often considered enteric bacteria and were common in GI sites. This biological contamination is likely related to animal excretion in GI. Of the taxa associated with common urban pollutants, Actinomycetales and Bacillales were notable members of both Technosols and natural urban surface soils. The Order Actinomycetes had the greatest presence in ETP and is associated with common urban pollutants, a GI design greater connectivity to urban runoff with smaller area to manage and retain the runoff (Rahman et al., 2002; Yu et al., 2018).

Among the ecosystem services provided by GI features, these data suggest that Technosol associated bacteria have the potential to utilize or break down urban contaminants in addition to TPH. This ecosystem service is valuable for management strategies of urban ecosystems to remediate chemical and oil spills as well as heavy metals, especially for long-term contaminated sites (Wilson et al., 1999; Ellis et al., 2000; Mishra et al., 2001; Delgado-Balbuena et al., 2016). Bacterial Orders in the top 10% of Technosol community that were enriched compared to urban surface soils, were RB41, iii1-15, Saprospirales, Cytophagales, Pirellulales, Opitutales, Anaerolineae__envOPS12. These bacteria are likely the initial description of a potential consortium that characterize the GI Technosol community through contributing to bioremediation or are indicating of contamination. The top two groups are RB41 (Class Chloroacidobacteria, Order Acidobacteria) and iii1-15 (Class Acidobacteria-6, Order Acidobacteria) and they are common in urban soils and are prominent in soils globally; though their enrichment and role in the context of bioremediation in GI Technosols is unclear (Fierer, 2017; Huot et al., 2017). Natural urban surface soils had a greater abundance in Myxococcales, which contribute to removal of trace organic contaminants, specifically uranium, and may additionally suppress unwanted organisms (Cardenas et al., 2010; Phan et al., 2016; Robinson et al., 2016). The increase in abundance of Cytophagales in GI Technosols may indicate some functional redundancy within the diverse bacterial community. Cytophagales have previously been documented as enriched in urban soils contaminated with crude oil and can metabolize heavy metal (e.g., Selenium) (de Souza et al., 2001), which also reinforces their potential functional role as a bioremediator within GI features (Mukherjee et al., 2017). Rhodospirillales had decreased abundance in the Technosols, however, their presence may still contribute to pollutant degradation, since soils with phenanthrene,n-octadecane and PAH pollution had thriving populations of Rhodospirillales (Ji et al., 2013; Jiao et al., 2016). In the GI Technosols studied here, the contaminant concentrations were most likely too low to drive a response. Additional indicators of bacterial community response to TPH contaminants may also be detected through the increased abundance of bacteria typically used to facilitate bioremediation; such as, *Corynebacterium*, *Flavobacterium*, and *Bacillus* species, as well as *Pseudomonas* and *Micrococcus* species because of their notable biodegradation of crude oil (Mishra et al., 2001; Rahman et al., 2002; Paul et al., 2005). Since overall TPH levels were low GI sites were not considered polluted. The consequence of the low levels of contaminants detected in the GI sites is likely why respective taxa for bioremediation were not abundant. The bacterial communities of Technosols at GI sites have limited evidence of known urban contaminants. Further understanding of the role of GI features for bioremediation in urban areas requires future studies focused on the enrichments of specific bacterial taxa and contaminants. The bacterial community of different GI designs are influenced by a combination of biogeochemical parameters and GI features. Future studies using shotgun metagenome sequencing would corroborate functional capabilities (e.g., nutrient cycling and bioremediation of contaminants) from these unique GI sites and facilitate comparing observations regarding the urban soil microbiomes across analysis platforms.

## Conclusion

Our description of the microbiome of urban GI features has improved our understanding of the factors influencing bacterial community composition and the role of GI features in an urban ecosystem. GI design that integrates connectivity to urban street runoff can be a key constraint on the bacteria present in Technosol communities; in contrast, they may not have overwhelming effects on microbial-mediated ecosystem processes. Biogeochemical parameters were not reduced in highly connected, highly engineered GI features. Therefore, design and management of GI features that support high levels of soil organic matter will be important for sustaining the functions of GI features in highly stressed urban environments. One important consideration is the maintenance of healthy plant communities as part of the management and sustainability of GI features and functions. Storm events and resulting urban runoff will have strong impacts to GI sites and bacterial community, particularly in ROWB, and further investigation can address specific differences between SSIS and ETP designs.

Future analysis of the microbial communities of GI features should include high resolution studies of storm events that generate urban runoff as well as investigate the impact of different vegetation cover. To continue illuminating the microbial “black box” of urban ecosystem processes, metagenomic sequencing coupled with stable isotope analysis can identify key genes specific to biogeochemistry as well as composition of GI microbial communities. Additional analyses will be essential for characterizing the specific ecosystem services of GI features such as the containment or bioremediation of urban contaminants.

## Author Contributions

TM, PG, ZC, and GL contributed to the conception and design of the study. JJ, JK, MD, AP, GL, and JM contributed to data collection. JJ and MD performed the analyses. JJ interpreted the analyses and wrote the first draft of the manuscript. JK and MD wrote sections of the manuscript. TM and PG contributed substantially to the revision of the manuscript. All authors reviewed the final manuscript and approved the submitted version.

## Conflict of Interest Statement

The authors declare that the research was conducted in the absence of any commercial or financial relationships that could be construed as a potential conflict of interest.
