# Supplementary material for: Green Infrastructure Design Influences Communities of Urban Soil Bacteria
Source: Front Microbiol. 2019 May 16;10:982. doi: 10.3389/fmicb.2019.00982 (PMC6531853; doi:10.3389/fmicb.2019.00982)
Supplement: Supplementary file 1 [file Data_Sheet_1.docx]

**Supplemental Description:**

**PCR reaction conditions**

The 16S rRNA gene V1-V3 variable region PCR primers 27F/534R with the forward primer linked to the sequencing barcode (8 basepairs). HotStarTaq Plus Master Mix Kit (Qiagen, USA) was used to complete 28 PCR cycles of initial denaturation at 94°C for 3 minutes, followed by cycles of 94°C for 30s, 53°C for 40s and 72°C for 1min, then a final elongation step at 72°C for 5min.

After amplification, PCR products were checked in 2% agarose gel to determine successful amplification. Samples were pooled together in equal proportions based on their molecular weight and DNA concentrations. Pooled samples were purified using calibrated Ampure XP beads then were considered completely prepared to submit to the Illumina MiSeq as the project DNA library. All DNA sample preparation was completed, and the laboratory methods conveyed by Scot Dowd at Molecular Research Labs, Shallowater, TX.

**Supplemental Table 1.** Summary of biogeochemical measurements completed for each GI sample (Deeb et al. 2018).

| **Biogeochemical Measurement** | **Units** | **Microbial Connection** | **Reference** |
| --- | --- | --- | --- |
| Soil Organic Matter Content (OM %) | mg kg^-1^ | Soil organic matter is highest in the top 20-30cm of the soil and it functions as a main energy source for soil microorganisms | (McGeehan and Naylor 1988; Sinsabaugh, Hill, and Follstad Shah 2009) |
| Microbial biomass C content | mg C kg^-1^ soil | Measure of carbon within the living elements of soil organic matter (Bacteria, Fungi, Archaea) | (Bohlen et al. 2001; Jenkinson and Powlson 1976; Gonzalez-Quinones et al. 2011) |
| Microbial biomass N content | mg N kg^-1^ soil | Measure of nitrogen within the living elements of soil organic matter (bacteria, fungi, archaea) | (Jenkinson and Powlson 1976) (Anderson and Domsch, 2010) (Bohlen et al. 2001) |
| Potential net  N mineralization | µg N g dry soil^-1^ day^-1^ | Microorganisms breakdown organic nitrogen into ammonium to increase plant available nitrogen, as part of the nitrogen cycle | (Bohlen et al. 2001; Schimel and Bennett 2004) |
| Potential net nitrification | µg N g dry soil^-1^ day^-1^ | Microorganisms metabolize ammonium and produce nitrate as a by-product | (Bohlen et al. 2001; Martinez-Porchas et al. 2014) |
| Microbial respiration | µg C g^-1^ soil day^-1^ | Conversion of soil organic C to CO_2_ by soil microorganisms | (McGeehan and Naylor 1988; Bohlen et al. 2001) |
| Soil Organic Carbon  (SOC%) | mg kg^-1^ | Microorganisms break down soil organic C for energy utilization | (McGeehan and Naylor 1988) |
| Total Nitrogen  (N total %) | mg kg^-1^ | The total nitrogen content of the soil | (McGeehan and Naylor 1988) |
| Total Petroleum Hydrocarbons (TPH) | mg kg^-1^ | Certain microorganisms can degrade hydrocarbons, aiding in bioremediation efforts | (EPA method 8015-B)(Nikhil et al. 2013) |

**Supplemental Table 2.** Sequence count for each sample following significant stages in the analysis pipeline. After demultiplexing the samples, the average number of sequences for a sample was 72,345. Following the assignment of sequences as OTUs the average number of sequences for a sample were 60,828. Finally following the removal of non-target sequences there was an average of 59,263 sequences remaining for analysis of community composition and diversity.

| Site | Demultiplexed | OTU binned | Non-Target Filtered |
| --- | --- | --- | --- |
| GI.0.27 | 67980 | 54656 | 53984 |
| GI.3.2 | 72688 | 61747 | 60847 |
| GI.3.6 | 72269 | 60302 | 59094 |
| GI.7.9 | 69515 | 59259 | 57778 |
| GI.0.8 | 65314 | 54858 | 54092 |
| GI.0.14 | 54560 | 45278 | 44702 |
| GI.3.1 | 54032 | 46440 | 45713 |
| GI.4.5 | 61276 | 51139 | 49953 |
| GI.5.2 | 73456 | 62587 | 61749 |
| GI.10.4 | 71930 | 59977 | 58361 |
| GI.0.3 | 60707 | 50409 | 48918 |
| GI.8.18 | 69810 | 59038 | 58204 |
| GI.8.19 | 78218 | 66215 | 65291 |
| GI.8.20 | 73234 | 58572 | 57457 |
| GI.11.25 | 180076 | 158844 | 148163 |
| GI.12.26A | 71923 | 58696 | 54856 |
| GI.12.26B | 62072 | 52353 | 51424 |
| GI.12.26C | 73561 | 61521 | 60506 |
| GI.0.11 | 67733 | 57033 | 55922 |
| GI.0.21 | 60518 | 51050 | 50297 |
| GI.0.24N | 66198 | 54352 | 53517 |
| GI.0.24S | 64522 | 53883 | 52975 |

**Supplemental Figure 1.** Street side view of GI sites. ROWB sites (ETP and SSIS) have selected tree species also indicated for each site.

**
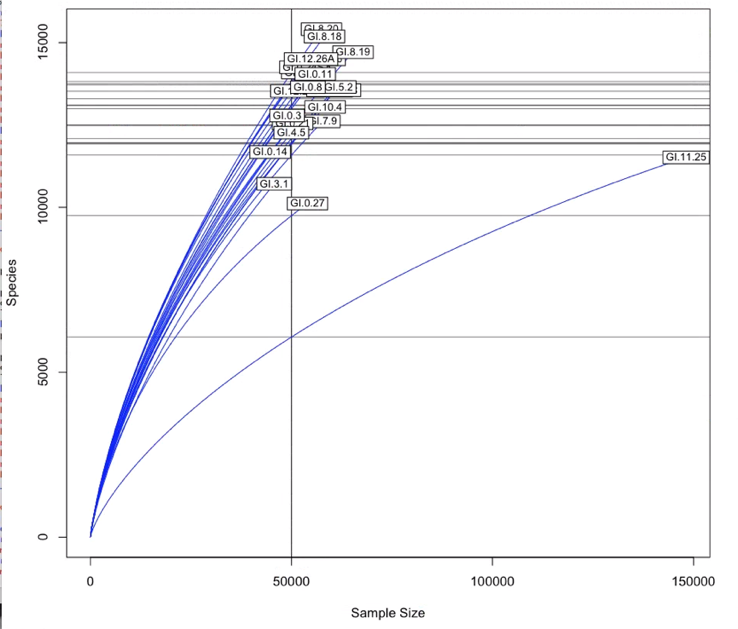

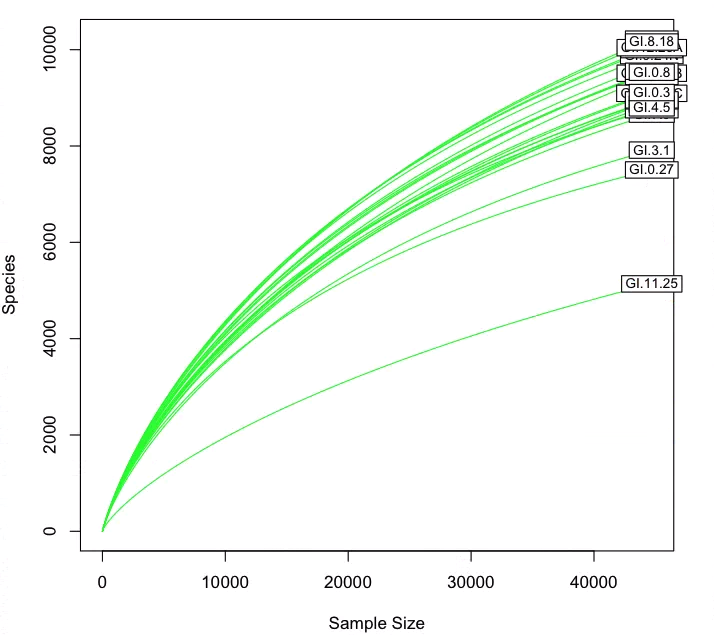
**

**Supplemental Figure 2.** Rarefaction curves calculated before and after rarefaction to the lowest sequence count for a sample (44,702). Curve was made as steps of 10 with 50,000 samples.

**Supplemental Figure 3.** Boxplot of alpha diversity metrics for GI sites. The range represented by the box is lacking for ETP1 and UF because of no replicates. The outlier for VS sites (G1.11.25) is evident as it is the lowest point for all metrics.

**Supplemental Figure 4.** Correlations between biogeochemical properties and the top bacterial Orders for each GI design. Red indicates positive correlation and Blue indicates negative correlation, intensity of the color corresponds with the strength of the correlation. Significance (p<0.05) is represented with (*).

**A.**
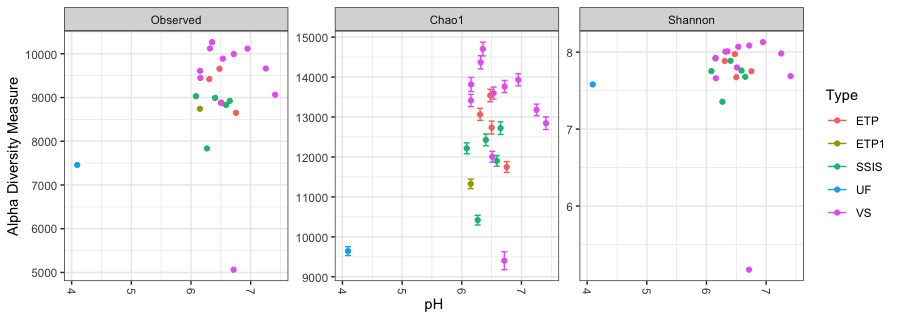


**B.**
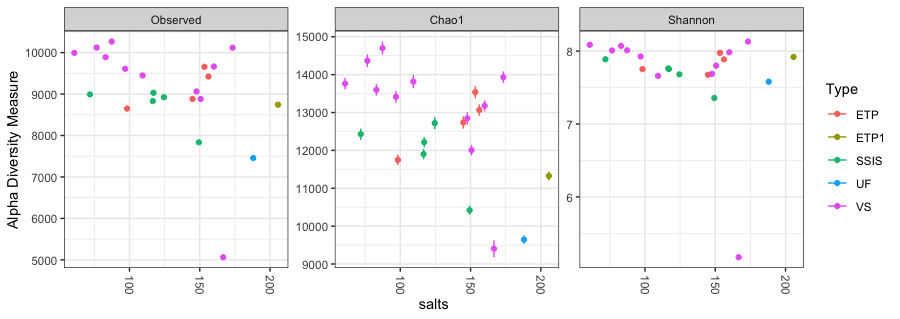


**C.**
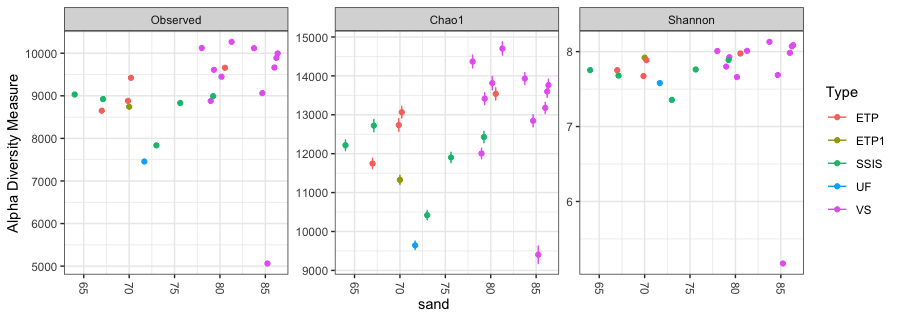


**D.**
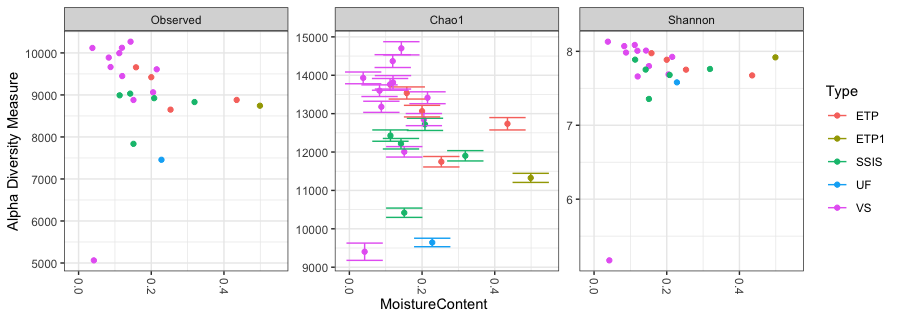


**E.**
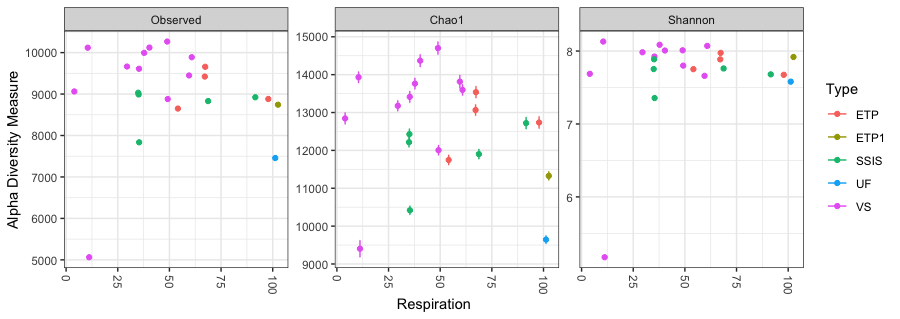


**Supplemental Figure 5.** Microbial diversity associated with additional measured biogeochemical parameters. **A.** pH **B.** salts **(mg kg^-1^)** **C.** sand (%) **D**. Moisture Content (%) **E.** Respiration (µg C g^-1^_dry soil_ day^-1^).


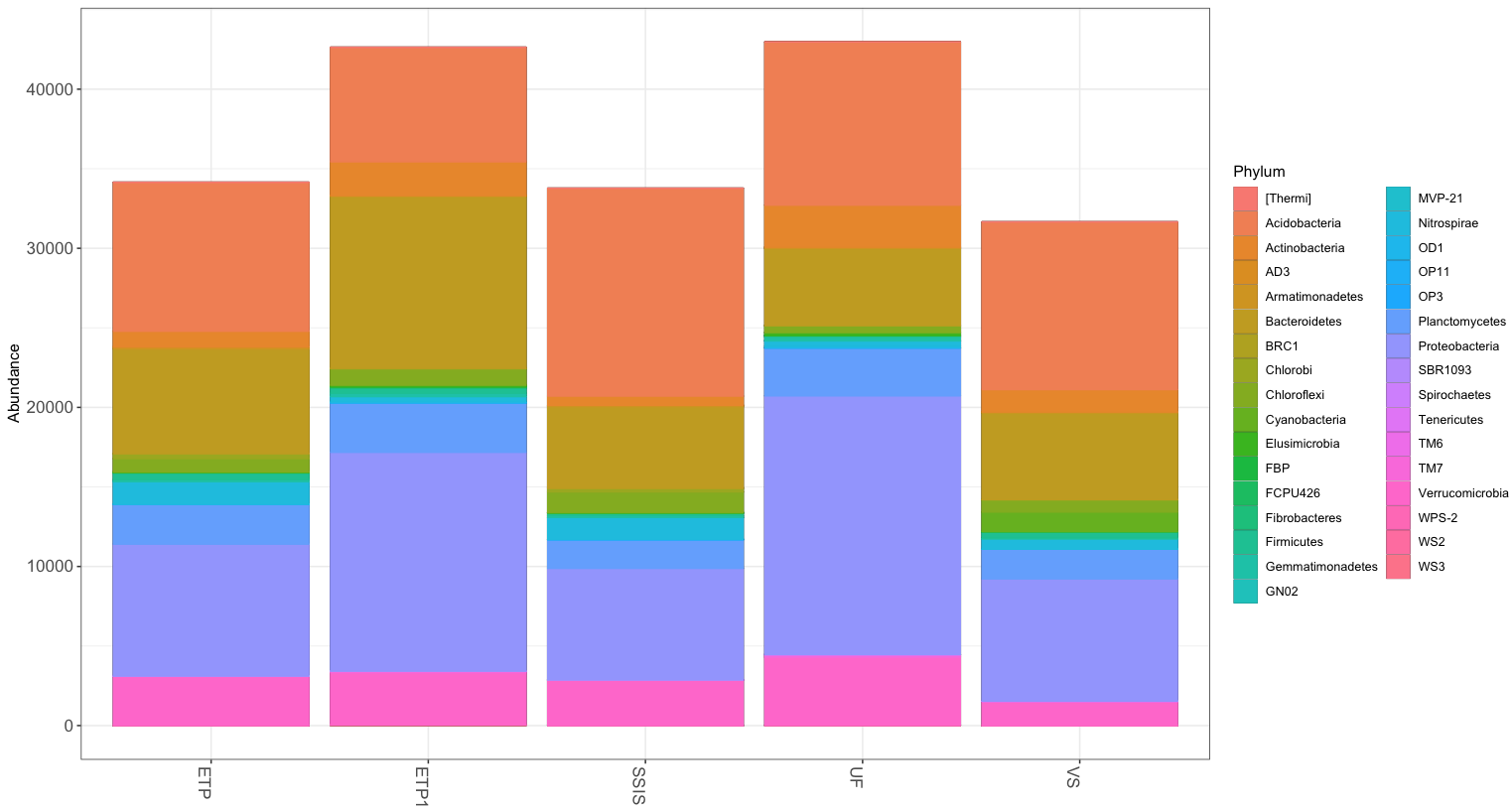


**Supplemental Figure 6.** Bacterial community of Technosols of each GI design represented as the number of sequences for each Phyla (N=35).
